# Lessons for Theory from Scientific Domains Where Evidence is Sparse or Indirect

**DOI:** 10.1007/s42113-024-00214-8

**Published:** 2024-12-03

**Authors:** Marieke Woensdregt, Riccardo Fusaroli, Patricia Rich, Martin Modrák, Antonina Kolokolova, Cory Wright, Anne S. Warlaumont

**Affiliations:** 1 Department of Cognitive Science and Artificial Intelligence, Radboud University, Nijmegen, The Netherlands; 2 Donders Institute for Brain, Cognition, and Behaviour, Radboud University, Nijmegen, The Netherlands; 3 Language and Computation in Neural Systems, Max Planck Institute for Psycholinguistics, Nijmegen, The Netherlands; 4 Department of Linguistics, Cognitive Science and Semiotics, School of Communication and Culture, Aarhus University, Aarhus, Denmark; 5 Interacting Minds Center, School of Culture and Society, Aarhus University, Aarhus, Denmark; 6 Linguistic Data Consortium, University of Pennsylvania, Philadelphia, PA USA; 7 Department of Philosophy, University of Bayreuth, Bayreuth, Germany; 8 Institute of Microbiology of the Czech Academy of Sciences, Prague, Czech Republic; 9 Second Faculty of Medicine, Charles University, Prague, Czech Republic; 10 Department of Computer Science, Memorial University of Newfoundland, St. John’s, NL Canada; 11 Department of Philosophy, California State University Long Beach, Long Beach, CA USA; 12 Department of Communication, University of California, Los Angeles, Los Angeles, CA USA

**Keywords:** Explanation, Evidence, Cognitive science, Theory development, Theoretical virtues, Underdetermination

## Abstract

In many scientific fields, sparseness and indirectness of empirical evidence pose fundamental challenges to theory development. Theories of the evolution of human cognition provide a guiding example, where the targets of study are evolutionary processes that occurred in the ancestors of present-day humans. In many cases, the evidence is both very sparse and very indirect (e.g., archaeological findings regarding anatomical changes that might be related to the evolution of language capabilities); in other cases, the evidence is less sparse but still very indirect (e.g., data on cultural transmission in groups of contemporary humans and non-human primates). From examples of theoretical and empirical work in this domain, we distill five virtuous practices that scientists could aim to satisfy when evidence is sparse or indirect: (i) making assumptions explicit, (ii) making alternative theories explicit, (iii) pursuing computational and formal modelling, (iv) seeking external consistency with theories of related phenomena, and (v) triangulating across different forms and sources of evidence. Thus, rather than inhibiting theory development, sparseness or indirectness of evidence can catalyze it. To the extent that there are continua of sparseness and indirectness that vary across domains and that the principles identified here always apply to some degree, the solutions and advantages proposed here may generalise to other scientific domains.

## Introduction

In several research areas, empirical evidence for the theories at play is mostly sparsely or indirectly available. Evidence is *sparsely available* insofar as there is not much data relevant to inferences about the theories under consideration, or if that data is distributed such that it is hard to find. For example, engravings and other proto-symbolic traces produced by early humans are important data for understanding the evolution of human cognition and communication, but such traces are usually not preserved and remote, to the point where there is a 30,000-year gap between prior and new tracings that provide evidence of humans producing abstract drawings (Henshilwood et al., 2018). Evidence is *indirectly available* insofar as more inferential steps are required to get to a theory or conclusion. For instance, precursors of human language (e.g., gestural or vocal proto-signs) do not fossilise at all, and therefore they cannot be directly observed by researchers working to understand their evolution. Instead, researchers must make inferences from what can be observed—such as anatomical changes in the vocal tracts of ancient hominids and extant primates and experimental findings about modern humans developing novel communication systems—hence relying more heavily on background assumptions and adjacent theories (about early humans’ relation to other primates and to modern humans, how biological and cultural evolution work, etc.).

In principle, the sparsity and indirectness of evidence are two separate concerns. However, they are closely related. On one hand, what we count as evidence depends on what inferences we can make based on the available data; hence, when there is little direct evidence, even if there is ample data, evidence for a theory may be effectively sparse. On the other hand, the sparser the evidence is, the harder it may be to establish strong theories about what the evidence tells us, without also making strong assumptions; hence, sparse evidence may tend to be effectively less direct.

Reliance on sparse and/or indirect evidence puts scientists in a difficult position.^1^ When philosophers of science discuss what makes a good theory, *evidential accuracy* is often the first theoretical virtue to be mentioned: for a theory to be good, it has to fit the empirical evidence well (also known as *accuracy*, *empirical adequacy*, or *empirical fit*) (Tulodziecki, 2021; Keas, 2017; McMullin, 2013). Douglas (2009) categorises empirical adequacy as a minimal criterion (along with *internal consistency*, which requires that the theory contain no contradictions), which is more fundamental than other familiar theoretical virtues such as *simplicity*. Schindler (2022) shows that scientific practitioners generally agree, collectively ranking evidential accuracy second in terms of importance (after internal consistency) out of six theoretical virtues. But what if empirical evidence is only indirectly or sparsely available for the theory being developed? Guest (2024) argues that when evaluating theories, an important criterion is to consider its *empirical interface*: That is, the potential it has to make explicit the relationship between theory and observation, and how these two influence each other. Following this, we argue that one can work with sparse and indirect evidence, but given that the gap between theory and observations is substantial, one should take special care to articulate how theory and evidence do (or don’t, or potentially could) fit together.

In this light, we discuss a case study of a research domain in which empirical evidence is particularly sparse and indirect: the evolution of human symbolic communication—a key feature of human cognition. The kinds of evidence used to support theories in this domain are normally indirect (because we cannot directly observe how mental states, gestures, and sound types evolved). This indirect evidence is often sparse as well, since past data has not always been preserved archeologically and even extant species to which humans can be compared are limited in number, relatedness to modern humans, and observability. Here, indirectness and sparseness of evidence are pervasive challenges unlikely to be completely solved by technological innovation in measurement methods.

Scientists studying the evolution of human symbolic communication have taken various approaches to grapple with these challenges, such as using computational modelling, performing experiments on modern humans, and translating concepts, insights, and findings from adjacent theories and domains. From this case study, we distill five virtuous practices supporting theory development especially when empirical evidence is indirect and/or sparse. These five practices are ways of articulating the gap between theories and observations. In doing so, we follow Guest ’s (2024) call upon scientific practitioners to characterise and examine the criteria (“virtues and vices”) they use to *adjudicate* between theories, and engage in *metatheoretical* dialogue about these. The five virtuous practices we distill here can be viewed as a step in that direction for the subfield of cognitive science that does research on the evolution of human cognition.

Although this paper focuses on a particular research domain, indirectness and sparseness of evidence certainly also apply in many other domains of cognitive science and may be more widespread than initially supposed. Indirectness of evidence may go unnoticed because the data processing, operationalisations, and related assumptions that take us from raw data to evidence are taken for granted (Flake & Fried, 2020; Fried et al., 2022). Sparseness of evidence can also go unnoticed; for example, there may be a wealth of data, but the signal within that data may be so subtle that *relevant* evidence is still sparse (Woodward, 2010). (This issue has been extensively discussed for neuroimaging methods; see Klein 2010; Wright 2018, 2021; Aktunc 2014; Roskies 2008, 2010).

Thus, the five virtuous practices we distill in this paper may contribute to developing good theories in general. The case of extreme sparseness and indirectness of evidence and the focus on a particular scientific domain simply provide a particular viewpoint for considering what the virtuous practices could be and giving examples for how they can be put into practice. Further, note that we do not argue that all five virtuous practices we propose should be normatively applied by all studies that aim to develop theory to explain cognitive or other phenomena for which empirical evidence is sparse or indirect. The different virtuous practices we propose contribute to theory development in different ways, as we explicitly discuss below. Depending on the type of phenomenon under investigation, the current state of scientific understanding of that phenomenon, the type of theory, and the available methodologies, some of these virtuous practices may be more important, or more feasible, than others.

The paper is structured as follows: First, the “Underdetermination and Evidence” section discusses the relation of sparseness and indirectness of empirical evidence to theory development. Then, the “Five Virtuous Theory-Building Practices Drawn from Human Symbolic Communication Evolution Research” section discusses in turn each of the five virtuous practices that we propose. These are (i) making assumptions explicit, (ii) making alternative theories explicit, (iii) computational and formal modelling, (iv) establishing external consistency with related theories, and (v) triangulation across different forms and sources of evidence. Per virtuous practice, we first discuss examples from our case study on the evolution of human symbolic communication, from which we distill that practice as playing an important role in theory development when evidence is sparse and indirect. We follow up each of these case study examples with a discussion of the merits of the distilled virtuous practice in more general terms. Finally, in the “Discussion” section, we close with a discussion of how we see the role of these five virtuous practices in theory development in general.

## Underdetermination and Evidence

### Underdetermination

Theory development is strongly driven by evidence. At first blush, then, having only sparse and/or indirect evidence might seem to irreparably undermine theory development. The evidence may not clearly favor particular theories, or it may only do so under further assumptions which are themselves contested.^2^ It is important to recognise, though, that this state of affairs is not as exceptional as it may seem. In fact, it is related to the problem of *underdetermination*, which has been extensively studied in philosophy of science (see Douven 2013, for a review). Theories are underdetermined if the evidence available does not establish what we are to believe about them. While underdetermination can be formulated in different ways—contrastively, holistically, alethically, etc.—and is not uncontroversial, it has been persuasively argued that a moderate level or amount of underdetermination holds for all theories at all times. That is, there are always logical gaps between data, theory, and evidence. In other words, for evidence to favour one particular theory over another, additional assumptions are always needed (for example, that we have operationalised the theoretical constructs in meaningful ways, that measurement is adequate, etc.). Multiple forms of reasoning, assumptions, and evidence are used to bridge the gap (Nelson, 2022; Harding, 1976; Baghramian, 2013).

That we cannot decisively establish theories from evidence alone is not, by itself, a reason to avoid developing theories; it just means that further resources need to be used. When evidence is sparse or indirect, then, we need to rely even more on these other resources. The theoretical virtues alluded to in the Introduction are among these resources: We may favor a theory based on a virtue such as simplicity or external consistency. Along these lines, Guest (2024) proposes that we zoom out from the classical theoretical virtues and develop a *metatheoretical calculus* for determining what a virtuous theory consists of (see also Guest and Martin 2023).

There can be further strategies for developing and evaluating theories, which are suited to specific contexts, such as that of cognitive science. For example, recent meta-scientific work in cognitive science has called for the need to formalise theories as full causal explanations and test them against *theoretical* plausibility constraints first, *before* testing them against empirical evidence (Adolfi et al., 2024; van Rooij & Baggio, 2021, 2020), a practice also advocated for in computational modeling (Gelman et al., 2020).

Regarding theory development with sparse or indirect evidence, then, there are three important points: Firstly, the fact that our evidence is insufficient to decisively adjudicate over theories is nothing special; it is the normal case in science. We simply need to attend more to other resources when the evidence runs short. Secondly, extra-evidential resources are important for all science; hence our recommendations in this paper may have value even for scientists whose evidence is less sparse and indirect. Thirdly, by providing recommendations for an extreme case of limited evidence, we can more clearly highlight principles that should be applied to other cases as the context makes it appropriate.

### Non-Identifiability and Weak Identifiability

Non-identifiability is a related statistical inference concept that is relevant for thinking about the impact that sparse and indirect evidence have on theory development. A parameter in a statistical model is considered *non-identifiable* if multiple different values of the parameter result in exactly the same distribution of all observable quantities included in the model (Greenland, 2011). If a parameter of scientific interest happens to be non-identifiable, theorists know that appealing to more data cannot completely answer their scientific question or resolve their problem. Thus, evidence may be effectively sparse even when highly detailed records of observations are available. In less extreme cases—i.e., those of *weak identifiability*—the data contain some information about the parameter; however, changes in the parameter make such a small difference to the distribution of the observable quantities that theorists cannot in practice learn anything useful about the parameter from the amount of data that are or could plausibly be available (see, e.g., Gelfand and Sahu, 1999 or Auger-Methe et al., 2021 for a more formal discussion). The typical example is (near) collinearity of predictors in a linear model, such that data do not let us disentangle the contributions of two factors to an outcome as the two factors co-occur throughout the data.

In this way, many cases of theoretical underdetermination can be viewed from this statistical perspective, where a key parameter needed to distinguish a set of candidate theories is non-identifiable based on the observable data.^3^ Given a statistical model corresponding to a research question, one type of problematic sparseness and/or indirectness of evidence would be when a parameter of interest is (weakly) non-identifiable. Thus, in theory development, one should consider what impacts different kinds of evidence would have and whether non-identifiability or weak identifiability is an issue. In those cases, additional practices beyond more data collection may be especially important.

## Five Virtuous Theory-Building Practices Drawn from Human Symbolic Communication Evolution Research

One research area within cognitive science in which the issue of indirect and sparse evidence is immediately apparent is the evolution of human cognition, and particularly symbolic behaviour, including language and other communicative systems. The evolutionary origins of human cognition and symbolic behaviors are the subject of much debate. Compared to other animals, the human species is peculiar in its cultural products and processes. Humans have developed complex technology, political systems, other cultural practices, art, science, and for a large part created our own habitats (Heyes, 2012). These developments are made possible by cognition, cooperation, and culture (Jablonka & Lamb, 2006). Furthermore, some of the cognitive abilities that have made these developments possible (such as sophisticated social cognition and language) have been argued to be (at least in part) a result of cultural evolution themselves, meaning that they have accumulated over generations through social learning, rather than genetic inheritance (Heyes, 2018a; Woensdregt et al., 2021).

However, research on human symbolic communication evolution faces severe issues of sparseness and indirectness of evidence. Put simply: Cognitive processes do not fossilise, and neither do the brain, gestures, signs, or spoken words. Therefore, direct evidence for particular cognitive abilities cannot be found in the archaeological record. The evolution of language has even been argued to be “one of the hardest problems in science”, as we only have indirect traces of it (Christiansen & Kirby, 2003). But many researchers interested in human cognition and symbolic behaviors are also interested in their origins. They have creatively employed a broad range of methods and data sources (e.g., computational modelling, experimental studies with adult humans, neuroimaging, longitudinal observations of human children, and comparative observations across extant species) to acquire indirect evidence that can be used to develop and assess theories of language evolution and related topics.

From the research that is being done in this area, we distill five practices that researchers use to deal with sparseness and indirectness of evidence. These are (i) making assumptions explicit (“Making Assumptions Explicit” section), (ii) making alternative theories explicit (“Making Alternative Theories Explicit” section), (iii) computational and formal modelling, (“Computational and Formal Modelling” section) (iv) external consistency with related theories (“External Consistency with Related Theories” section), and (v) triangulation across different forms and sources of evidence (“Triangulating Across Forms and Sources of Evidence” section). Below, we discuss each of these five practices in turn. Per practice, we first discuss how it has aided and can further enhance theory development regarding the evolution of human symbolic communication. We follow each of these up with a more generalised discussion of the virtuous practice’s broader value for aiding theory development, in the case of sparse and indirect evidence of varying degrees.

### Making Assumptions Explicit

#### Case Study

Research on the evolution of human cognition and symbolic behaviors avails itself of a wide variety of empirical evidence and assumptions, which are only sometimes made explicit. One approach to studying human communication evolution is to use experiments with modern humans to inform theories about cultural evolution in prehistoric ancestors. For example, when trying to identify the symbolic properties (or lack thereof) of early engravings from Blombos Cave and Diepkloof Rock Shelter in South-Africa (Henshilwood et al., 2018; Texier et al., 2010), experimental subjects were used as proxies to understand the cognitive affordances of those symbolic traces. These experiments suggest that these early engravings might have been used for aesthetic and group identity purposes more than for referential communication (a hypothesis already advanced in Hodgson and Pettitt 2018) (Tylen et al., 2020; Wisher & Tylen, 2024).

Other experimental work has focused on the enactment of cultural transmission chains in the lab (Caldwell et al., 2016; Miton & Charbonneau, 2018; Tamariz & Kirby, 2016; Tamariz, 2017; Caldwell & Millen, 2008; Muller & Raviv, 2024; Tamariz & Papa, 2023). For example, a process of cultural evolution can be simulated experimentally by creating a transmission chain of (pairs of) participants, who learn a behaviour by observing, or interacting with, participants from a previous “generation” in the chain. If the processing and reproduction of behaviors and symbolic resources are in any way constrained by participants’ cognitive system and activities, we could expect behaviors and signs to evolve to better fit these constraints. Such transmission chain experiments were used to assess the conditions under which patterns such as those in early engravings from Blombos and Diepkloof, or the structural characteristics of language, could have evolved (Tylen et al., 2023; Pagnotta et al., 2024; Perfors & Navarro, 2014; Tamariz & Kirby, 2016; Tamariz, 2017; Tamariz & Papa, 2023). These studies provide novel theoretical contributions by using innovative approaches to enable inferences about inaccessible processes.

The approach described above relies crucially on the assumption that data produced by modern humans provide legitimate evidence about early hominin cognition. In other words, they assume that the human cognitive system has not qualitatively changed over time, at least in those respects that the studies investigate. This is a strong assumption, given that, for instance, the human cognitive system undergoes extensive changes as it learns to read and write (Dehaene et al., 2015). Furthermore, they assume that those aspects are unaffected by the cultural particularities of the experimental participants. However, by specifying these assumptions, it becomes clearer that we should evaluate the plausibility of significant changes of different aspects of the system. Specifically, we could expect early visual processing components to be relatively stable over hominin evolution and scientists could in principle triangulate theories and evidence from comparative, cross-cultural, and computational studies to develop these ideas. Specifying assumptions explicitly is an essential first step in this process. While making assumptions explicit does not legitimise them, it does provide targets for further reflection, critique and follow-up research, specifying the inference and assessing our confidence in it as well as identifying what kinds of additional evidence could be useful to strengthen it.

Another general approach to studying the evolution of symbolic behavior is to observe the development of communicative behaviors in human infancy. Observations of the order in which foundational language capabilities emerge during ontogeny may inform theories regarding the phylogenetic order of emergence of those capabilities in the ancestors of modern humans. For exa-mple, infants show a prolonged period of vocal (or in the case of sign language learners, gestural) babbling that appears to lack referential meaning (Oller et al., 2013) and increases with age in its complexity and speech-likeness preceding the emergence of meaningful and intelligible words at around the first birthday (Warlaumont, 2020). This ordering of behaviors in ontogeny may reflect a “natural logic” (Oller, 2000): producing recognisable conventional words in the vocal modality logically depends on having precise voluntary control of the vocal tract musculature in a way that is “functionally flexible” with regard to the speaker’s current emotional state. These ontogenetic observations are potentially relevant for phylogene-tic theories because the same natural logic may also apply phylogenetically. Historically, however, the idea that ontogeny recapitulates phylogeny appears to be supported in some cases (e.g., Heldstab et al., 2020) and unsupported in others (e.g., Yang, 2013), and has occasionally been misused as justification for racist ideology and inhumane treatment of various groups of people (Varga, 2020). Thus, it is important to specify the precise reasons for assuming that phylogeny should resemble ontogeny in the particular case of vocal repertoire emergence, to be explicit about the degree of theoretical and empirical support for that assumption, and to pursue continued research to assess the validity of the assumption.

In the literature on the (cultural) evolution of human cognition and language, overt discussion of the assumptions of a given theory often occurs in reviews and philosophical papers that discuss or classify existing theories (see Acerbi and Mesoudi, 2015; Dunstone and Caldwell, 2018; El Mouden et al., 2014, for examples). In our experience, papers that present novel observational, experimental, or computational research often discuss their assumptions in more implicit terms. However, exceptions to this pattern provide examples of how explicit discussion of assumptions can clarify authors’ claims and enrich theory development. For example, there are computational modelling papers that include a dedicated (sub)section that explicitly summarises and/or critically discusses the assumptions of the model (e.g., Roberts and Levinson, 2017, pp. 414–415; Winters, 2019, pp. 11–12), and experimental papers that explicitly and critically discuss the assumptions of their experimental design (e.g., Silvey et al., 2019).

#### Broader Relevance

Our examples above showcase the first virtuous practice we propose: **making assumptions explicit** is important for developing theories when evidence is sparse or indirect. Indeed, doing so not only leads to more transparency, but also provides targets for theory evaluation and develops the nexus of external evidence. Beyond the above examples, theoretical assumptions play a role in the explanatory relationship between theory and evidence more generally. The Duhem-Quine thesis states that it is not possible to test a hypothesis in isolation, but only conditional on certain background assumptions (Quine, 1951; Harding, 1976). When a theory’s empirical prediction is shown to be false, it does not follow that the entire theory is in error: possibly, just one of the assumptions is false. The structure of theories includes a “hard core” of tenets that proponents are really committed to as well as peripheral assumptions that are needed to generate empirical predictions. These peripheral assumptions can be dropped and replaced if the theory is not successful enough empirically; in fact, improving them and promoting some to the “hard core” can be seen as progress for the theory (Lakatos, 1970; see Cooper, 2006, for an application to cognitive science.)

Therefore, properly evaluating a theory requires making its key assumptions explicit, including both fundamental assumptions and those which are only instrumental (see also Guest ’s, 2024, construct of *metaphysical commitment*). When doing so, it is important to articulate both the existing justification for, as well as the consequences of, these assumptions. Without knowing what would count as evidence for or against the assumptions, such that they can be evaluated independently, it is hard to assess a distribution of truth-values over them. Scientific disciplines concerned with causal inference from observational data, such as economics and epidemiology, are increasingly recognising and applying this virtuous practice, for instance promoting the formalisation and use of counterfactuals and causal graphs (specifically *directed acyclic graphs*; see also the “Making Alternative Theories Explicit” section). This literature provides inspiration for how to deal with evidentially-underdetermined theories by formalising their assumptions and requisites, possibly in comparative fashions.^4^

Finally, in addition to what assumptions *are* being made by a given theory, it can also be relevant to be explicit about what the theory remains agnostic about. Are there parts of the theory that can capture different subvariants? That is, that could be filled in with different specific assumptions that wouldn’t affect whether the theory holds?

### Making Alternative Theories Explicit

#### Case Study

When theories are underdetermined by evidence, it is often the case that many alternative theories are possible. A recent example in the study of the evolution of human cognition and communication pertains to the discovery of suggestive marks on a limestone pillar in the Rising Star cave in South Africa, where remains of *Homo naledi* have been found (Berger et al., 2023). The suggestion is that these markings might be intentional and might have been produced by *Homo Naledi*, thus potentially retro-dating the emergence of symbolic behaviors in hominins by more than 100,000 years, and to an earlier branch of the hominin family. The open peer reviews of the paper in which the findings are reported explicitly postulate different possible scenarios motivating these markings, thus exemplifying collective efforts in theory development. For instance, the markings could be (i) natural, (ii) produced unintentionally or (iii) produced at times for which we have no hominin remains, either earlier or more likely later than the suggested 224,000 to 335,000 years ago, for instance by later *Homo sapiens*. Each scenario has implications that cannot be tested given the available evidence at the time of the article; but they do provide foci for further theory development, and help identify which kind of evidence might be used in the future to further determine the theory.

Another example of alternative theories can be found in the debate on whether linguistic communication originated from gestural communication before transitioning to being primarily vocal, or whether precursors to language were primarily vocal from the beginning of the divergence from our non-human primate relatives. Owing to the lack of archaeological data distinguishing between these two general theories, this debate exemplifies a case where evidence is both sparse and indirect. What evidence there is mainly comes from observation of and experimentation with young children and non-human primates. Much of the evidence for the gestural origins hypothesis comes from findings that non-human primates, including great apes, show much greater voluntary control over gesture production than over vocalisation (Arbib et al., 2008; Clay & Genty, 2018; Genty et al., 2009, 2014; Liebal et al., 2014, chapter 5; Pollick & de Waal, 2007; Townsend et al., 2017). For example, attempts at teaching aspects of human language to individuals from these species have been much more successful in the gestural modality than in the vocal (Corballis, 2008; Meguerditchian, 2022). Additionally, in at least some cases, human infants appear to learn words more easily in signed versus spoken modality (this early advantage disappears at some point in the second postnatal year) (Meier, 2016). Furthermore, young children have been observed to use gestures to supplement early spoken word productions in place of using additional spoken word modifiers (Cartmill et al., 2014). Yet, human language today is predominantly vocal, and there appears to have been substantial biological adaptation for this modality, with vocal precursors to speech being present in high quantities even in the earliest stages of human infancy (Oller et al., 2019) as well as anatomical features of adult vocal tracts that appear to be adaptations related to speech production (de Boer, 2019).

While the debate remains unsettled, articulating these two contrasting theories has proven fecund. For instance, researchers motivated by this question have undertaken to make direct comparisons of vocalisation and gesture rates in human infancy, helping to establish the extent to which both modalities are used for various communicative functions, thus helping shed light on their potential roles as ontogenetic precursors (Burkhardt-Reed et al., 2021). The debate has also led to the development of alternative theories that propose multimodal (Frohlich et al., 2019; Levinson & Holler, 2014) and even multicausal (Prieur et al., 2020) origins. The more that these alternative theories can be made explicit and detailed, the more they can inspire new empirical research as well as further theoretical proposals. When the evidence is so sparse and indirect, this development of alternative detailed theories is clearly essential, because the space of possible accounts is so vast and creativity and insight regarding possible scenarios are needed to link them to current potential indirect sources of evidence.

Finding and articulating alternative theories is a responsibility that goes beyond individual researchers, to encompass scientific communities sharing and distributing this work across researchers (Solomon, 1992). A concrete example of how this process can be structured in a scientific community is provided by the Causal Hypotheses in Evolutionary Linguistics Database (CHIELD, https://correlation-machine.com/CHIELD/) (Roberts et al., 2020). The aim of this project—launched at the biggest conference of the Language Evolution scientific community (*Evolang*) in 2018 (Roberts, 2018)—is to collaboratively collect hypotheses about language evolution (including sets of alternative hypotheses that aim to explain the same phenomenon), with references to the literature in which those hypotheses are advanced.^5^ All hypotheses in this database are represented in the form of causal graphs (mostly *directed acyclic graphs*; DAGs) (for an introduction to DAGs, see Cunningham, 2021, chapter 3; McElreath, 2020, chapters 1 and 5). Causal graphs represent variables and constructs as nodes and the causal connections between them as arrows, thus providing a more intuitive overview of implicit assumptions and comparisons between theories (Tennant et al., 2021). Further, given a directed acyclic graph (DAG), there are formal rules for identifying possible confounds and missing information (see, e.g., Fusaroli et al., 2023; Fusaroli et al., 2024; Hunermund and Bareinboim, 2023). By providing an open source, collaborative, dynamic database of theories and hypotheses in the field of language evolution in a causal graph format, CHIELD allows more systematic exploration, evaluation, and integration of multiple theories into a coherent narrative (Krykoniuk & Roberts, 2024). For instance, if one paper establishes a connection between two variables (say, length of utterance and propensity to re-use one’s interlocutor’s words), suggesting that the one causes the other, one can explore the dataset to identify whether there are common causes of the two that explain their covariance (e.g., age) and determine, accordingly, which additional evidence should be produced to discriminate between the two possible theoretical constructs.

These examples highlight the importance of making alternative theories explicit. Not only do they issue alternative explanations that theories can be evaluated against, but they give insight into a theory’s assumptions, and can help guide further theory development.

#### Broader Relevance

The case studies above showcase the second virtuous practice we propose: **making alternative theories explicit**. Theories ought to be evaluated against other, competing theories (Capaldi & Proctor, 2008). Therefore, when proposing a given theory, it is important to explicitly discuss possible alternatives. What alternative scenarios would be compatible with the available evidence? And what evidence would be needed to favour one scenario over another? Furthermore, as Guest (2024) argues, it is important to be explicit about how the theory one puts forward is embedded within its field, among other theories, as well as about the theory’s *reach* (i.e., to what extent could it be applied outside the domain it was developed in?).

One may argue that this puts too much burden on individual researchers, and that it is the remit of others to generate alternative theories. However, highlighting alternative theories and competing ideas is an important scientific practice because it signals that scientists are operating under a specific governing norm: openness to challenges, falsification, and further theory development. The nature of science is such that researchers should doubt theoretical claims in the face of evidence that challenges them, and move on from a theory when it becomes clear that the preponderance of evidence disfavors it. Depending on the situation, this may involve improving the theory, developing an alternative, or adopting an already existing alternative. As a matter of human cognition and cognitive bias, it is sometimes difficult to pivot from or discard ideas that have a familiar or preferential history, or that are central to a theory that one has committed time and resources to. Nonetheless, science is ultimately an epistemic endeavor that aims at the production and dissemination of knowledge. That good scientific practice requires a certain degree of honesty and integrity would suggest the importance of acknowledging competing theories and the landscape of epistemic friction (Potochnik et al., 2018 p. 25). Further, identifying key alternatives might be crucial in formalising one’s own assumptions. Finally, our recommendations pertain to the collective enterprise of doing science and the distribution of burdens in the community (Solomon, 1992; Ellemers, 2021).

Another consideration is the possibility that multiple theories may be correct, i.e, “explanatory pluralism”. Indeed, a diversity of correct theories may even be desirable in order to achieve a more comprehensive understanding of scientific phenomena involving complex systems (Dale et al., 2009; McCauley & Bechtel, 2001). Cognitive neuroscience is replete with examples. One comes from the extensive work that is being done on the hierarchical predictive processing theory of the brain (PTB), especially on the free energy formulation of the theory. Using the activity of mesocorticolimbic dopaminergic (DA) systems as a case study, researchers have shown that various mature DA hypotheses vindicate a kind of explanatory pluralism, which provides both theoretical friction for attempts at unification as well as impetus for new theoretical development (Colombo & Wright, 2017; Fabry, 2017). While explanatory pluralism complicates the task of adjudicating between theories because multiple theories may be simultaneously correct without being equivalent to each other, it supports the value of simultaneously considering alternative theories and of making those theories and their assumptions as explicit as possible. Theories sometimes evolve over time in tandem by deploying conceptual advancements and findings in adjacent fields of inquiry and borrowing from each other; this involves a co-evolutionary research ideology, in which explanatory pluralism is a background assumption (McCauley, 1986).

### Computational and Formal Modelling

#### Case Study

Formal modelling has played a central role in the field of language evolution (Smith, 2014; Kirby et al., 2014; Kirby, 2017). When evidence is indirect and/or sparse, computer simulations become especially appealing. Investigating historical processes or phenomena on an evolutionary timescale is at best difficult and often just not possible; but researchers can hasten their progress by simulating how it might have gone. Computational models (often agent-based) allow researchers to specify their theories (Guest & Martin, 2021), manipulate different factors that play a causal role in their explanations, and explore the dynamics that ensue (Madsen et al., 2019). Researchers in this area are interested in explaining where the structural features that are shared across the world’s languages come from (Hockett, 1960; Wacewicz and Zywiczynski, 2015; Pleyer & Zhang, 2022), but also what factors may cause differences in structure (Evans & Levinson, 2009). Among the factors playing a role in such explanations are (i) the goals and constraints that define communicative interactions between agents, (ii) the cognitive/learning biases of agents, and (iii) influences on cultural transmission, such as who learns from whom, or how much input each learner receives. Through repeated cycles of use and learning, small changes to the language that cause it to better fit such goals, constraints, or biases can accumulate over cultural evolutionary time, and cause systematic structure in the model’s miniature languages to emerge (Smith, 2014; Kirby et al., 2014; Kirby, 2017).

Computational modeling has also been crucial in understanding how evolutionary changes in vocal tract morphology would affect vocalisation repertoires (de Boer, 2019). For instance, studies of the effects of the descent of the larynx (de Boer, 2010) and the loss of air sacs (de Boer, 2012) on the ability to produce differentiated vowel types has been greatly aided by computational models of vocal tract acoustics, which allow vocal tract shape to be connected with synthesised sounds that can then be analyzed acoustically and phonetically. Computer simulation of this type also enables the roles of vocal tract shape to be dissociated from neural motor control mechanisms, which is especially helpful in attempting to reconstruct human vocal evolution, given that evolution of vocal tract anatomy and neural mechanisms are correlated and not easily dissociated in the data that are available from extant primate species. Indeed, this approach has led to the conclusion that vocal tract adaptations for speech, while apparent, are not strictly necessary to produce a wide range of speech sounds, and thus, changes in neural motor control are more likely as the crucial difference between human vocal flexibility and non-human primates’ relative vocal inflexibility (Boë et al., 2002; Fitch et al., 2016).

Computational modelling can be proficuously combined with experimental work (for examples of such experimental work on cultural transmission chains, see Tamariz, 2017; Tamariz and Kirby, 2016; Miton and Charbonneau, 2018; Caldwell et al.., 2016; Caldwell and Millen, 2008; Tamariz and Papa, 2023).

An advantage of combining these experimental approaches with modern humans as subjects with computer simulations, is that the former provides an anchoring of the simulations into empirical human data, while the latter allows for scaling up to a broader range of contexts and timescales than experiments could possibly encompass (e.g., larger populations and more generations of cultural transmission), as well as to more easily assess the impact of specific cognitive features (e.g., by manipulating the working memory size of the agents, which could not be easily and ethically done in humans) (Galke et al., 2022).

#### Broader Relevance

The case studies above showcase the third virtuous practice we propose: **to pursue computational/formal modelling.** Formalising a verbal theory (for example in the form of a computational model or a causal graph) has many advantages (van Rooij & Blokpoel, 2020; van Rooij & Baggio, 2021, 2020; Guest & Martin, 2021; Smaldino, 2017; Guest, 2024). Two are of particular importance here. Firstly, formalising a theory allows it to be tested against theoretical plausibility constraints, for which empirical evidence is not necessary (Adolfi et al., 2024). For example, it is important to analyse whether a proposed explanation of a cognitive process is tractable, because human minds can only compute tractable functions, and theories postulating intractable functions are thus a priori implausible (Van Rooij, 2008; van Rooij & Baggio, 2021; van Rooij et al., 2012). Secondly, formalising a theory can help specify what would count as evidence in favour of the theory (Guest, 2024). For instance, theorists might use computer simulations to see what patterns of data the theory predicts. Indeed, computational models may be especially valuable in the case of complex systems, where system behavior is nonlinear and often chaotic (de Boer, 2006; Smaldino, 2017; Arthur, 2021) or when experiments are not feasible (Mitchell, 2009). Analogously, a causal graph formalisation (specifically in the form of a *directed acyclic graph*; DAG) can reveal plausible confounds—e.g., common causes or colliders—in reasoning about the theory, and identify which kind of observations and/or experiments would be needed to assess the presence of a causal link. Simulations on the causal graph can also be used to assess how different levels of confound would impact the findings, thus better qualifying inferences even in absence of the optimal experiments (Greenland, 1996).

Connecting this virtuous practice to the virtuous practice of making assumptions explicit, formalising a theory (e.g., in the form of a computational model) also forces the modeller to be explicit about their assumptions (Guest & Martin, 2021; Smaldino, 2017), because a full computational model cannot be formalised, let alone implemented, while leaving key assumptions implicit or vague. In complex systems, initial conditions and precision can have a drastic effect on the unfolding behavior of a system (Lorenz, 2000; Klower et al., 2023), so descriptions of computational models should contain as much relevant detail as possible about them, both aiding understanding and facilitating reproducibility. In the case of agent-based models, standardised formats are advanced to help researchers clearly and completely describe all aspects of their models to aid transparency and reproducibility (Grimm et al., 2010, 2006). In addition to good practices for reporting computational models in publications, openly sharing the (well-documented) code of model implementations is vital (Janssen et al., 2020), but this has to be done with care and cannot come in the place of clear and complete model specification (Cooper & Guest, 2014). Furthermore, to reap the full benefits of computational modelling for clarifying assumptions, Cooper and Guest (2014) argue that modellers should be explicit about what assumptions in the model are *theory-relevant*, and what assumptions are theory-irrelevant (e.g., just necessary for implementing the model, but not part of the theory itself^6^) (see also McCloskey, 1991). For example, the structure of a causal graph for causal inference would usually be considered theory-relevant, but to implement a full model, we may further assume that the encoded relationships are linear which would usually be theory-irrelevant.

While simulations can be a very powerful tool, we also want to note that they must be used and interpreted with care, and that there are unsettled questions surrounding what exactly simulations can provide. We highlight a few such questions here. First, since computational models can be used to run computer simulations, some have argued that such simulations can be seen as a form of experimentation (Parker, 2013) or even a distinct source of evidence (Currie, 2018, chapter 9). It is controversial whether framing the role of simulation in these ways is appropriate, though (see Lehtinen and Kuorikoski, 2021, for an overview and discussion). While it seems clear that the results of simulation studies can be informative, the nature of the evidence they provide differs from that of a conventional experiment. Second and relatedly, what kind of explanation can simulations provide? This question is particularly pressing for theorists interested in etiological explanations. It has been proposed that while simulations cannot tell us how a natural phenomenon actually arose, they may instead be able to tell us “how-possibly” it arose. Simulations are a relatively new tool in scientists’ toolkit, and discussion of how they could provide “how-possibly” explanation (and what its epistemic value would be) is ongoing (see Frey and Šešelja, 2018, for discussion). A third concern with computer simulations is that they may be used to produce almost any result (Windrum et al., 2007; Lehtinen & Kuorikoski, 2021). Consequently, it is important to prioritise relatively simple and transparent simulations that are strongly motivated by theory (Hartmann, 1996), and to pay special attention to the robustness of the results (Muldoon, 2007). Note that various computational models deal with emergent phenomena that are poorly understood prior to simulation under different parameter values and initial conditions (Miller & Page, 2007).

These types of formalisation are especially valuable in situations where much of the system of interest is unobservable, and thus evidence is particularly sparse or indirect or otherwise difficult to adduce (Mitchell, 2009; Greenland, 1996). Formal models enable different possibilities regarding unobservable aspects of the system to be explicitly proposed, facilitating productive discussion within the discipline and across related theories (Roberts et al., 2020; Fusaroli et al., 2024). Such models also help researchers to notice when competing theories cannot be distinguished by the available evidence.

### External Consistency with Related Theories

#### Case Study

When theorising about the evolution of cognition, evolutionary biology is a salient related field. Thus, researchers studying the (cultural) evolution of cognition have often sought to establish external consistency with theories from evolutionary biology and other related fields. For example, theory development on cultural evolution (where behaviours change as they are passed on over generations through social learning) has taken inspiration from constructs and processes that have been considered of causal importance in evolutionary biology, such as *variation*, *transmission*, and *selection*. However, whether such theoretical constructs can be mapped from one domain to another has to be considered very carefully (Guest, 2024). In the case of cultural evolution, social learning obviously works differently from genetic inheritance: cultural traits are created and transformed by human minds, with cognitive and learning biases influencing the changes being made to behaviours or artifacts, with the goals and constraints of the cultural trait also having influence on the process of change (Gabora, 2019; Heyes, 2018b; Perry et al., 2021; Creanza et al., 2017; Chellappoo, 2021; Ingold, 2007). This difference between social learning and genetic inheritance has led to much debate on, for example, the relative importance of the concepts of replication versus reproduction in cultural evolution, and whether stability in cultural transmission has to come from faithful “copying” of behaviours, or whether it can also result from transformation by minds that have similar biases or contexts that have similar needs (Miton, 2023; Morin, 2016; Acerbi & Mesoudi, 2015; Mesoudi, 2016). Thus, while theories from evolutionary biology have been helpful in providing ideas and frameworks for theories of cultural evolution, the analogy is imperfect and there must be careful discernment about which specific aspects of biological evolutionary theory are and are not applicable to cultural evolution. Another reason to be cautious in prioritising external consistency between these two adjacent theories is that evolutionary biology itself is subject to recent criticisms and calls for urgent reform (Tanghe et al., 2018; Baedke, 2021; Zeder, 2018; Rose, 2009).

External consistency can also take the form of consistency of a target theory with adjacent theories that form some of the assumptions of the target theory. The long debate about whether and how descriptions of group selection can legitimately play a role in explaining features of human cognition—such as our pronounced capacity to cooperate—provides another example of how establishing external consistency with related theories is potentially valuable yet non-trivial (Okasha, 2001; Richerson et al., 2016). Much of this literature has been overwhelmingly theoretical, using “evidence” from computer simulations, if at all (see the “Computational and Formal Modelling” section for a discussion of the nature of such evidence). However, by exploiting external consistency, it has been possible to incorporate specific assumptions into theorising on this topic. For example, work by Bowles and Gintis (2013) incorporates our current knowledge about early human social life—e.g., group size, frequency of conflict, and population exchange—into their models of the evolution of cooperation, to reduce the space of possible outcomes and infer more specific and better-supported conclusions. Similarly, we can appeal to evidence about modern people’s cognitive capacities (e.g., our ability to keep track of others’ beliefs and behaviours; Waade et al., 2023; O’Grady et al., 2015) to further support arguments about cooperation in our ancestors.

It is important to re-iterate that researchers must be careful with external consistency; seeking fit with adjacent theories also requires re-evaluating those other theories as appropriate, asking whether we (still) believe them or whether the state of the art has been updated. For example, new evidence for neuroscientific or bioacoustical theories can change which assumptions we should rely on in our theories regarding the evolution of human symbolic communication; developments in those related scientific fields may require updating our theories. Evolution is a phenomenon about which it is easy to project biases or problematic ideology about humans and what is natural or unnatural. Evolutionary psychology is controversial for many reasons, including disagreement about whether it takes over sexist assumptions (see, e.g., Levy, 2004; Liesen, 2007; Cameron, 2015; Grossi et al., 2014; Kelly, 2014). It is also the case that the majority of published research on human psychology, behavior, neuroscience, etc. has been heavily biased toward samples that are neither representative of the diversity of human cultures and living conditions around the world nor of living conditions over the course of hominin evolution. This limitation should be remembered if relevant for theories that aim to explain aspects of the evolution of human cognition and communication (Henrich et al., 2010; Kline et al., 2018; Muthukrishna et al., 2021). Yet, uncovering biases and history as having affected related theories can be a helpful prompt to consider the extent to which related biases and history may be at play in unduly shaping the focal theory (Guest, 2024). It is thus especially important, in the case of theories about the evolution of cognition, to periodically re-evaluate their external consistency, and whether the ways in which external consistency is realised are appropriate.

#### Broader Relevance

The examples above showcase the fourth virtuous practice we propose: **to seek external consistency with theories of related phenomena**, including theories from other domains. This means endorsing a standard theoretical virtue that appeared on Kuhn’s (1977) list of theoretical virtues. As McMullin (2013, p. 503) writes, *‘A theory will almost inevitably depend in part on other related theories; the stronger their warrant, the better its own case’.* Hence, it should count in favor of a given theory that it fits well with other accepted theories only insofar as we really think we can trust those theories; this might not be the case, for example, if those other theories reflect problematic biases (Longino, 1996). External consistency is thus an especially important relational property when a theory must rely less on empirical evidence; critically drawing on neighboring theories can supply not only theoretical support but possibly even new empirical premises or assumptions. When there is a paucity of data, or when the support it provides to a given theory is highly indirect, a theory may be unable to directly ground itself in evidence. Consequently, it becomes all the more important for researchers to stabilise the theory and maintain its motivation. Seeking external consistency with theories of related phenomena is one way to do this; theorists can bracket the problem of sparse or indirect evidence and instead make salient the logical relationships to other valid and relevant theories.

### Triangulating Across Forms and Sources of Evidence

#### Case Study

Preceding sections have shown that insight into the evolution of symbolic and communicative behaviour in hominins can come from many different sources. Scott-Phillips and Kirby (2010) propose four aspects of the evolution of language that are useful to distinguish, as they imply different sources of evidence (see also Roberts et al., 2020; Smith, 2018). The first aspect is *pre-adaptation*: the preconditions for a language ability, often associated with biological evolution and genetic inheritance. The second is *co-evolution*: the ways in which the early communication systems of our hominin ancestors and these pre-adaptations influenced each others’ evolution. The third is *cultural evolution*, a process by which linguistic structure first emerged, through repeated learning and use over generations (where the means of transmission over generations is not genetic inheritance, but social learning, in which cognitive biases play a role). Finally, the fourth aspect is *language change*: the ongoing changes in language that remain observable today, over relatively short timespans (starting from as little as several decades, for example by comparing different generations of language users). Although these aspects were initially described for language evolution, they apply to any form of behaviour that is subject to cultural evolution (i.e., transmitted and adapted over generations through social learning). Research questions about the first three of these aspects all deal with sparse and indirect evidence. We argue that different sources of evidence can be triangulated to build support for theories that address these various aspects of language evolution, mitigating some of the sparseness and indirectness of evidence (cf. Irvine et al., 2013).

For example, the archaeological record gives insight into early hominin ways of living, technologies, and group sizes, from which inferences can be made about the underlying cognition and culture (Currie & Killin, 2019; Romano et al., 2020).^7^ For example, stone tools from the Palaeolithic period provide a relatively continuous record of technological advancements in our hominin ancestors, which in turn give insight into their cognitive abilities and social lives. Stout (2011) analysed the hierarchical action sequences that are required to create the stone tools found in different Palaeolithic time periods, showing that compared to earlier tool types (Oldowan), later tool types (Acheulean) rely on more complex hierarchical action planning, and probably teaching (see also Caldwell et al., 2017). Teaching in turn is arguably enhanced by language and metacognitive abilities (Dunstone & Caldwell, 2018; Brandl et al., 2023; Laland, 2017; Lombao et al., 2017). The archaeological record can then be supplemented and/or corroborated by genomic and linguistic studies, giving a different view on (i) population size and bottlenecks via coalescent theory (Nordborg, 2019; Rosenberg & Nordborg, 2002), (ii) group migration via the study of biological ancestry, and (iii) (for later time periods) cultural transmission via the study of linguistic ancestry (Jager & Wahle, 2021; Greenhill, 2015).

The combination of experiments and computational models can also be a powerful approach to gain converging support for a theory. As discussed in the “Case Study section, experiments with modern-day humans can provide insight into the cognitive constraints and biases that may have shaped language and other symbolic behaviour, but only on the assumption that the relevant cognitive factors have not changed over many tens of thousands of years. One such form of experimentation involves transmission chains, intended to simulate a process of cultural transmission (Miton & Charbonneau, 2018; Caldwell et al.., 2016; Tamariz & Kirby, 2016; Tamariz, 2017; Caldwell & Millen, 2008; Tamariz & Papa, 2023). For example, returning to the cognitive and social processes necessary for making Palaeolithic stone tools, Cataldo et al. (2018) used transmission chain experiments to explore how different means of transmission affected participants’ performance in creating Oldowan-type stone tools through flintknapping. They found that participants’ performance improved when teaching was allowed (as opposed to an imitation/emulation condition in which pupils could only observe their tutors but not interact with them). Furthermore, they found that performance improved even more when the teaching involved language, but the latter effect seemed to be caused mostly by the beneficial effects of gesture, more so than speech. As discussed in the “Case Study” section, agent-based modelling can also be used to simulate such processes, allowing researchers to manipulate the cognitive make-up of the agents, the ways in which the agents interact, and the environment in which they interact (e.g., adding different selection pressures). The computational models can explore scenarios that are not possible with modern humans and the human subjects experiments can validate the computational modeling approach.

One consideration regarding triangulation across sources of evidence for developing theories of the evolution of human cognition and communication is the availability of that detailed evidence for access by other scholars. Sufficient detail is necessary to ensure comparisons are based on comparable measurements and definitions; a lack of availability of protocols and raw video or audio samples can make triangulation across sources of evidence, such as across observations from different animal species, extremely difficult. Open science practices, including sharing datasets (videos, audio, images, and other data types), simulation code, and analysis code can make triangulation and integration across sources much more feasible. For instance, the creation and dissemination of the CHild Language Data Exchange System (CHILDES, MacWhinney, 2000) provides a wealth of samples of child-adult conversation data across ages, languages, and clinical conditions. CHILDES data has enabled computational researchers to build models of language learning using real child-directed adult language as input (MacWhinney, 2010). It also enables comparison of model performance to that of real children. This has informed debates about the extent to which various aspects of language can in principle be learned by a general prediction-driven learning algorithm. This in turn bears on the question of the role of domain-general adaptations in language evolution (Lewis & Elman, 2001),^8^

#### Broader Relevance

The case studies above illustrate the fifth and final virtuous practice we propose: **to triangulate across different forms and sources of evidence.** Triangulation can take different forms (Carter et al., 2014), but we understand it to involve studying a single question or phenomenon through multiple distinct means (see, e.g., Campbell and Fiske, 1959; Denzin, 2007). (Kuorikoski and Marchionni, 2016, p. 227) defend the practice and argue that the value of triangulation lies in *“controlling for likely errors and biases of particular data-generating processes”*. Plausibly, evidence that is sparse and/or indirect is especially likely to mislead us or to be misinterpreted, which makes such controls especially valuable.

There are excellent prospects for productive triangulation in many contexts with sparse evidence. In such contexts, we are typically interested in phenomena that are causally important and thus have left marks on the world we observe (Currie, 2018). It is hard to know what specific words our hominin ancestors used for “rock”, but that is not causally important. Instead, what is causally important for understanding the origins and evolution of human symbolic communication is to find out what cognitive and socio-interactive abilities enabled humans to refer to things using arbitrary signs, as well as the impact that such communication might have had on cognition and socio-material practices. The changes in human brains, cognition, and cultural practices that allowed the evolution of language should have left *multiple traces:* e.g., in the non-linguistic abilities of the hominins and thus the material remnants they left (both in terms of material culture and of population dynamics and settlements), in brains of *Homo sapiens*, and potentially in our genome. Analogously, the use of symbolic communication might have allowed unprecedented forms of coordination, thus enabling different population dynamics, and the development of practices such as cave-painting or tool-making which require complex socially transmitted skills, as well as complex logistics to produce and store the necessary materials and support the artists or toolmakers during production (Wisher et al., 2023; Stout, 2011). Hence, we can triangulate by looking for these multiple traces and checking whether they all suggest, or are compatible with, a common cause.

The process of triangulation will usually involve a complex dialogue between the theory under investigation, midrange or bridging theories, and evidence. Currie (2018) proposes the term “exquisite corpse modelling” meaning a careful, reflexive combination of various, often imperfect sources of evidence that can bear upon the problem at hand (*exquisite corpse* referring to the game in which several people contribute to, e.g., a drawing, while only being allowed to see the end of the other person’s contribution). On the one hand, limited availability of evidence shapes researchers’ ability to discern details of a theory and may force them to reduce their epistemic aims. In the other direction, a well-developed theory could suggest additional sources of possible evidence and motivate the development of new midrange theories that would enable the incorporation of this additional evidence (see also Dale et al., 2023). Finally, we could realise that some known theory has the potential to work as a midrange/bridge theory for our problem. For example, Anderson et al. (1947) noticed that the theory of cosmic rays, combined with the theory of radioactive decay, predicts that all carbon in living organisms should be slightly radioactive and that this radioactivity should decay in dead matter without contact with the atmosphere, leading to the development of radiocarbon dating. This effectively established radioactive decay as a mid-range theory connecting measurements of radioactivity in archaeological finds with claims about their age and thus a large body of theories describing historical processes.^9^

## Discussion

Philosophers of science have long debated the inventory of theoretical virtues, their relationships to the goodness of a theory, and their role in theory choice. One of the virtues that is often given primary importance is *evidential accuracy* (also known as *accuracy*, *empirical adequacy* or *empirical fit*) (Tulodziecki, 2021; Keas, 2017; McMullin, 2013; Douglas, 2009; Schindler, 2022). However, some research areas within cognitive science develop theories about processes and phenomena for which empirical evidence is only sparsely or indirectly available. In this paper, we looked at practices used to deal with sparse and indirect evidence in one such research area: the evolution of human communication and symbolic behaviour. From this case study, we distilled five virtuous practices that can aid theory development when evidence is sparse or indirect. These are (i) making assumptions explicit, (ii) making alternative theories explicit, (iii) computational and formal modelling, (iv) establishing external consistency with related theories, and (v) triangulation across different forms and sources of evidence. Next, we discuss some qualifications regarding these practices and the extent to which they may be useful across disciplines.

The role of theories in cognitive science is to help us explain and understand cognitive phenomena, and it has been argued that a greater emphasis on theory development will be essential for progress in cognitive science research (Goldrick, 2022; van Rooij & Baggio, 2020, 2021). Much of the current discourse on reforms in psychological and social research focuses on pre-registered confirmatory research (Chambers, 2017): given existing findings or theory-driven predictions, can we falsify them adhering to strict pre-imposed methodological constraints, ensuring the rigor of the procedure? The five virtuous practices we put forward here emphasise an orthogonal perspective: abduction (instead of deduction), that is, the cumulative construction of only hypothetical and at least partially underdetermined theories. Each of the five virtuous practices we put forward in this paper helps in the process of expressing, exploring, and evaluating such theories.

As discussed in the Introduction, we submit that the five virtuous practices put forward here should be seen as complementary practices that can aid theory development, without thereby being stringently normative. Which of these virtuous practices are most important or most feasible to implement will depend on the research area, type of question, and type of theory. While the evidence is particularly obviously sparse and indirect when it comes to questions regarding the evolution of human cognition and communication, evidence in cognitive science and other fields is rarely if ever complete and perfectly direct. We therefore believe that the value of the virtuous practices we propose may reach beyond cases where empirical evidence is obviously highly sparse or indirect, providing potential lessons for theory development in general.

## Data Availability

Not applicable

## Footnotes

1 Sparseness of evidence is not always problematic. In some cases, relatively few data points may be required to decide between competing theories. For example, there are special situations in which theoretical competition can be resolved by an *experimentum crucis*; in these cases an abundance of data is unnecessary because even limited data will be decisive. We can consider these to be cases where evidence is not actually sparse relative to the quantity and density of evidence needed to decide between theories, in contrast with the typical situation in our case study, where evidence *is* sparse relative to what is needed to support or reject a theory regarding the evolution of symbolic communication.

2 Examples include the inability to distinguish between serial and parallel processing from completion times (Townsend, 1972) and underdetermination of theories of representation of visual imagery by behavioural data (Anderson, 1978).

3 In some publications, the terms “non-identifiability” or “unidentifiability” are fully synonymous with “underdetermination”. Here, however, we will use “non-identifiability” only in the statistical sense, while we use “underdetermination” in a more general sense, including cases that are less quantitative in nature, or where no explicit statistical model is available.

4 Assumptions can also play a role in resolving the identifiability problems in statistical models we discussed in the “Non-Identifiability and Weak Identifiability” section: A parameter can be made identifiable by introducing additional assumptions.

5 Read more about this “Causal graph challenge” that was launched at the *Evolang* 2018 conference here: http://www.replicatedtypo.com/the-evolang-causal-graph-challenge/11903.html.

6 Note that this distinction is reminiscent of the distinction between a theory’s hard core of assumptions and peripheral assumptions (see the “Making Assumptions Explicit” section); but they are not the same. Importantly, a theory’s peripheral assumptions at a given time are part of the theory at that time. In contrast, a model used to develop a theory will typically include many assumptions that are not part of the theory at all. The theorist knows that they are false, but uses them to keep the model simple or tractable. See the distinction between *substantial*, *Galilean*, and *tractability* assumptions in Kuorikoski et al. (2010).

7 Taking this further, *Material Engagement Theory* builds on non-computational, embodied and enactive theories of cognition, to propose a view on cognitive archeaology in which mind and materiality are inextricably intertwined (Gallagher & Ransom, 2016; Iliopoulos, 2019; Malafouris, 2019; Malafouris & Koukouti, 2017; Malafouris, 2020). Following this theory, cognition and its evolution should have left even more of a trace in the material world (and vice versa).

8 Another example of such an open dataset is D-PLACE: A Global Database of Cultural, Linguistic and Environmental Diversity (Kirby et al., 2016) although statistical analysis on such a dataset should be done very carefully to guard against spurious correlations (Roberts & Winters, 2013).

9 Triangulation can also help resolve identifiability problems in statistical models (see the “Non-Identifiability and Weak Identifiability” section). For example, a model may be extended to incorporate a new type of data that is sensitive to changes in the parameter of interest (see, e.g., section 5.9 of Gelman et al., 2020 or Auger-Methe et al., 2021 for examples and more discussion).
